# Rupture of Hepatic Artery Aneurysm as a Cause of Severe Upper Gastrointestinal Bleeding and Cholangitis in a Liver Transplanted Patient

**DOI:** 10.5152/tjg.2024.24039

**Published:** 2024-08-01

**Authors:** Cumali Efe, Ersin Batıbay, Osman Yüksekyayla, Mehmet Kolu

**Affiliations:** 1 Department of Gastroenterology, Harran University, Şanlıurfa, Türkiye; 2 Department of Radiology, Harran University, Şanlıurfa, Türkiye

Dear Editor,

A 65-year-old man who presented with hematemesis, melena, and intermittent febrile episodes was admitted to the hospital. He had undergone living donor liver transplantation due to HBV-related cirrhosis 7 years earlier and had performed serial percutaneous transhepatic biliary drainage and endoscopic retrograde cholangiography (ERCP) for biliary stricture. The last PTBD followed by ERCP were performed 6 months ago for the management of biliary stricture at another medical center. On admission, his blood pressure was 95/60 mm Hg and his heart rate was 115 beats/min. Physical examination revealed yellow discoloration of the body and tenderness in the right upper quadrant without guarding. Abnormal laboratory results were as follows: leukocyte count 14 700/mm^3^ (neutrophils 78%), hemoglobin 9.4 g/dL, hematocrit 29, alanine aminotransferase 268 IU/L (0-55), aspartate aminotransferase 242 IU/L (0-34), alkaline phosphatase 374 IU/L (64-164), gamma-glutamyl transferase 235 IU/L (0-37), total bilirubin 6.5 mg/dL (0.6-1.2), and CRP 62 (0-5 mg/dL). These laboratory markers were not abnormal at the examination 6 months prior . A gastroscopy revealed fresh blood in the stomach and duodenum, but the exact bleeding source was not found. Subsequently, endoscopy was performed with a duodenoscope, which showed bleeding from the papilla of Vater (Figure 1A).

We performed triple-phase computed tomography that revealed a left hepatic artery aneurysm with a 25-mm diameter (Figure 1B). An ERCP showed significant dilatation in the common bile duct (CBD). A large amount of bloody matter was removed with a balloon sweep, and a plastic stent (10 Fr/10 cm) was inserted into the CBD to relieve cholangitis. Consecutively, hepatic artery angiography confirmed an aneurysm in the left hepatic artery (Figure 1C). The hepatic artery aneurysm (HAA) was successfully managed under radiological guidance using platinum coils, and blood flow from the left hepatic artery to the liver was observed (Figure 1D). Following these interventions, the patient’s melena resolved, bilirubin levels became normal, and he was discharged without any complications.

Hepatic artery aneurysms are the second common visceral artery aneurysms and are classified as true aneurysms and pseudoaneurysms.^1,2^ Common causes of HAA include abdominal trauma, hepatobiliary surgery, atherosclerosis, infections, malignancies, and percutaneous hepatic or biliary interventions.^2-4^ Our patient had 2 of the above-mentioned risk factors for the development of HAA. Although it is a very rare clinical condition, rupture of HAA should be considered among the differential diagnosis of upper gastrointestinal bleeding, especially in the presence of concomitant obstructive jaundice. Our case also highlights that thorough patient history as well as strong collaboration between gastroenterology and radiology are essential to reach a definitive diagnosis and prompt treatment of patients presenting with gastrointestinal bleeding due to rupture of HAA.

## Figures and Tables

**Figure 1.**
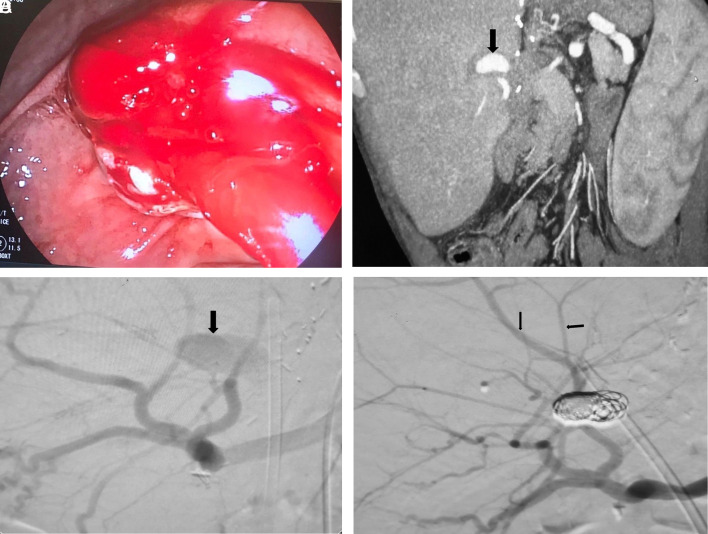
(A) Endoscopy showing active bleeding from the duodenal papilla. (B) Computer tomography image shows a pseudoaneurysm of the left hepatic artery. (C) Angiography image shows blood flow from the left hepatic artery aneurysm to the bile duct. (D) The coil embolization of the left hepatic artery pseudoaneurysm was successfully performed.
